# *EPHB2 *germline variants in patients with colorectal cancer or hyperplastic polyposis

**DOI:** 10.1186/1471-2407-6-145

**Published:** 2006-06-01

**Authors:** Antti Kokko, Päivi Laiho, Rainer Lehtonen, Sanna Korja, Luis G Carvajal-Carmona, Heikki Järvinen, Jukka-Pekka Mecklin, Charis Eng, Johanna Schleutker, Ian PM Tomlinson, Pia Vahteristo, Lauri A Aaltonen

**Affiliations:** 1 Department of Medical Genetics, Molecular and Cancer Biology Research Program, P.O. BOX 63, 00014 University of Helsinki, Finland; 2 Laboratory of Cancer Genetics, Institute of Medical Technology, University of Tampere and Tampere University Hospital, 33014 Tampere, Finland; 3 Molecular and Population Genetics Lab, London Research Institute, Cancer Research UK, 44 Lincoln's Inn Fields, London WC2A 3PX, UK; 4 The Second Department of Surgery, Helsinki University Central Hospital, P.O. BOX 262, 00029 Helsinki, Finland; 5 The Department of Surgery, Jyväskylä Central Hospital, Keskussairaalantie 19, 40620 Jyväskylä, Finland; 6 Genomic Medicine Institute, Cleveland Clinic Lerner Research Institute, Dept of Genetics Case Western Reserve University School of Medicine, 9500 Euclid Avenue, NE-30, Cleveland, OH 44195, USA

## Abstract

**Background:**

*Ephrin receptor B2 *(*EPHB2*) has recently been proposed as a novel tumor suppressor gene in colorectal cancer (CRC). Inactivation of the gene has been shown to correlate with progression of colorectal tumorigenesis, and somatic mutations have been reported in both colorectal and prostate tumors.

**Methods:**

Here we have analyzed the *EPHB2 *gene for germline alterations in 101 individuals either with 1) CRC and a personal or family history of prostate cancer (PC), or 2) intestinal hyperplastic polyposis (HPP), a condition associated with malignant degeneration such as serrated adenoma and CRC.

**Results:**

Four previously unknown missense alterations were observed, which may be associated with the disease phenotype. Two of the changes, I361V and R568W, were identified in Finnish CRC patients, but not in over 300 Finnish familial CRC or PC patients or more than 200 population-matched healthy controls. The third change, D861N, was observed in a UK HPP patient, but not in additional 40 UK HPP patients or in 200 UK healthy controls. The fourth change R80H, originally identified in a Finnish CRC patient, was also found in 1/106 familial CRC patients and in 9/281 healthy controls and is likely to be a neutral polymorphism.

**Conclusion:**

We detected novel germline *EPHB2 *alterations in patients with colorectal tumors. The results suggest a limited role for these *EPHB2 *variants in colon tumor predisposition. Further studies including functional analyses are needed to confirm this.

## Background

The Wnt signaling pathway plays a central role in the development of colorectal cancer (CRC). In the majority of cases the early events in tumorigenesis involve inactivation of the tumor suppressor gene *APC *and stabilization of β-catenin [1,2]. The constitutive activity of β-catenin/T cell factor-4 (Tcf-4) –complex leads to transcription of growth promoting genes, which together with subsequent inactivation of tumor suppressor genes drive the tumorigenesis further and enable the formation of abnormal growth patterns.

One of the direct transcriptional targets of β-catenin is *Ephrin receptor B2 *(*EPHB2*), whose protein product mediates the bi-directional migration and correct positioning of the cells along the crypt axis in the intestinal epithelium [3]. The gene is located in chromosomal region 1p36, which is frequently altered in many types of cancer [4]. Recent findings have demonstrated that *EPHB2 *appears to have a role in tumor progression, and acts as a tumor suppressor gene in colorectal cancer. In the study by Batlle *et al*. (2005) EPHB2 activity was observed to suppress CRC progression as the disruption of EphB2 in *Apc*^*min*/+ ^mice was shown to accelerate tumor formation and growth in colon and rectum [5]. In addition, premalignant lesions of the colon were shown to express high levels of EPHB2, whereas the expression was reduced in more advanced tumor stages [5]. Somatic mutations in *EPHB2 *have been observed both in colorectal and prostate tumors, and an *EPHB2 *germline mutation has been associated with an increased prostate cancer (PC) risk [6-8]. Huusko *et al*. (2004) identified a nonsense mutation Q723X in a PC cell line, which led to the identification of other novel missense (R199H, A297S, D679N, T909M), nonsense (K1019X), frameshift and splice site mutations in prostate tumors obtained from patients in USA and Switzerland [7]. In a later study by Kittles *et al*. (2005) the nonsense mutation K1019X was found to increase risk for PC over two-fold in an African American hereditary PC sample set [8]. In 2005, Alazzouzi *et al*. observed frequent frameshift mutations on the A9 repeat of the *EPHB2 *gene in primary colorectal tumors in patients of mostly Caucasian origin (Finland, Germany, Spain and Japan) [6].

In our recent study we found decreased *EPHB2 *gene expression and protein levels in tumors with serrated CRC when compared to tumors from patients with conventional CRC [9]. While no somatic *EPHB2 *mutations were observed in the serrated CRC tumors, one patient was found to harbor a germline *EPHB2 *alteration. This, together with the growing evidence of the role of *EPHB2 *in CRC tumorigenesis, prompted us to further investigate the role of *EPHB2 *in colorectal tumor predisposition. In this study, we have analyzed the *EPHB2 *gene for germline alterations in 101 patients with either 1) CRC and personal or family history of PC or 2) hyperplastic polyposis (HPP), a condition believed to be associated with serrated CRC [10].

## Methods

### Patient material

Altogether 101 normal tissue samples were collected from medical institutions in Finland, UK and USA from patients with 1) CRC and either personal or family history of PC (n = 63, mean age of onset 69 years, range 30–85 years) or 2) hyperplastic polyposis (n = 38, mean age of onset 46 years, range 16–69) (Table 1). The CRC patients belong to a well characterized and previously described population based series of 1042 Finnish CRC patients [11,12]. Family history of PC was considered positive if the patient had a first degree relative diagnosed with PC. HPP was diagnosed if there were at least three but typically over 10 histopathologically verified hyperplastic polyps. If less than five polyps were observed, at least two of them had to be large (>1.0 cm). The UK patients had between 10 to over 100 HPs, and the mean age of onset was 43 years (range 16–79 years). Five patients had one or more large HPs (range 1–3 cm), two had serrated adenomas, one had mixed adenomatous/hyperplastic polyps, three had adenomas and one had juvenile polyps. Family history of polyps or cancer was detected in 10% of these individuals, and 40% of these had developed CRC. Patients from the US had in general over 30 HPs, the mean age at HPP diagnosis was 51 years (range 19-68 years), and all but one had been diagnosed with serrated adenomas. In two cases there was history of HPP in the family. The two Finnish HPP patients were diagnosed with multiple hyperplastic polyps and serrated adenomas at 44 and 41 years of age.

The frequencies of the observed *EPHB2 *variants were further analyzed in either Finnish familial prostate (n = 164, mean age of onset 63 years, range 40–86 years) and colorectal (n = 159, mean age of onset 65 years, range 27–90 years) cancer patients, or in additional UK HPP patients (n = 40, mean age of onset 54 years, range 23– 74), respectively. The Finnish CRC patients belong to the aforementioned population based series of CRC patients [11,12]. The familial PC cohort has also been previously well characterized and described [13,14]. In addition, 282 samples from anonymous Finnish blood donors and 200 healthy UK individuals served as population-matched controls, respectively.

Patient information and samples were collected after obtaining informed consent. The study was approved by the ethics review committee of the Hospital District of Helsinki and Uusimaa (Dnr. 133/E8/03).

### Mutation analysis

Mutation screening of the *EPHB2 *gene [GenBank: AF025304] in the 101 normal tissue samples was performed by direct sequencing (ABI PRISM 3100 Genetic Analyzer, Applied Biosystems, Foster City, CA). All coding exons with at least 70 nucleotides intronic sequence at the exon-intron boundaries, except exon 1, were sequenced. Primer sequences and PCR conditions are available upon request. Loss of heterozygosity (LOH) was analyzed by comparing sequences from the normal and the corresponding tumor DNA at the site of a variant. Frequencies of the observed alterations in familial CRC, PC and HPP patient samples, as well as in healthy population-matched controls, were determined by either direct sequencing or DHPLC (variant R568W). The DHPLC analysis was performed using the Agilent 1100 Services DHPLC Instrumentation (Agilent, Palo Alto, CA) with HPLC Column 50 × 3.0 mm × ¼" Helix DNA (Varian, Palo Alto, CA), Helix BufferPak DNA Analysis buffers "A" and "B" (Varian), and the following parameters: T = 64°C, flow 0.4 ml/min, gradient 3 min 58–66% B-buffer.

## Results

Altogether four heterozygous germline missense changes, all previously unreported, were observed in the initial *EPHB2 *mutation screening of 101 samples (Table 2). An I361V (1081A>G) alteration in exon 5 was detected in a Finnish patient diagnosed with a microsatellite stable (MSS) rectal cancer at the age of 75 years and PC at the age of 73 years. The variant was not present in 164 familial PC patients, in 139 of 159 familial CRC patients (data from 87% of cases) or in 239 of 282 healthy population-matched controls (data from 85% of cases). The corresponding tumor tissue displayed loss of the wild type allele. This residue resides in the fibronectin type III domain, which is in the extracellular part of the protein and participates in cell-surface binding. Another missense change, R568W (1702C>T) in exon 9, was detected in a Finnish patient diagnosed with a MSS colon cancer at the age of 69 years. The patient's father, whose carrier status is unknown, had been diagnosed with PC at the age of 82 years. The variant was not observed in 164 familial PC patients, in 145 familial CRC patients (data from 91% of cases), or in 279 healthy Finnish controls. No loss of the wild type allele was observed in the colon tumor tissue. The residue is located in a juxtamembrane segment before a kinase domain in the intracellular part of the protein. The third missense alteration, D861N (2581G>A) in exon 14, was detected in a UK patient diagnosed with HPP at the age of 58 years. A review of her clinico-pathologic history revealed that, to date, she has had serrated adenomas and over 100 pancolonically distributed sessile HPs. The patient's mother, from whom no sample was available, had died of CRC at the age of 36 years. The variant was not observed in the additional 40 HPP patients or in 200 healthy UK controls. Due to the unavailability of tumor tissue DNA, LOH could not be determined. The variant is located in the intracellular protein kinase domain of EPHB2. The fourth change, R80H (239G>A) in exon 3, was initially observed in a Finnish patient whose MSS colon cancer was diagnosed at the age of 73 years. The patient's brother had been diagnosed with PC at the age of 60 years, and her father had died of gastric cancer at the age of 57 years. The variant was also found in 1/106 (0.9%) familial CRC patients and in 9/281 (3.2%) healthy population-matched controls. No loss of the wild type allele was observed in the tumor tissue. The variant is situated in the N-terminal ligand-binding domain at the extracellular part of the protein.

## Discussion

Ephrin receptors constitute a large family of receptor protein tyrosine kinases that together with ephrins, their membrane-bound receptor-like ligands, participate in developmental processes requiring organized patterning and movement of cells, such as axonal guidance, signal transduction, cell morphogenesis and angiogenesis [15,16]. These proteins are widely expressed in adult tissues with organ specific patterns [17]. In the intestine the most prominent ephrin receptor is EPHB2 [17]. Recently, the loss of EPHB2 expression has been associated with colorectal carcinogenesis. The reduction of the EPHB2 levels has been found to correlate with the degree of malignancy, as a significant decrease in number of EPHB2 positive cells and in staining intensity has been observed at the adenoma-carcinoma transition [5,18,19]. In our recent study, we found even more profound reduction in EPHB2 levels in colorectal tumors with serrated histology when compared to conventional CRC [9]. The link between reduced EPHB2 expression and poor survival has also been demonstrated [19]. In mouse studies, reduced Ephb2 activity has been shown to accelerate tumorigenesis in the colon and rectum, and to result in the formation of aggressive adenocarcinomas in *Apc*^*Min*/+ ^mice [3]. Based on the growing evidence of the role of EPHB2 in CRC, we hypothesized that *EPHB2 *germline alterations may predispose to colorectal tumors.

We found a total of four previously unknown *EPHB2 *germline alterations, of which three may associate with the disease phenotype. The corresponding tumor DNA was available from two variant carriers, and one of the tumors displayed loss of the wild type allele. All three alterations are located in amino acids showing conservation across species from mouse, rat, chicken and cow down to fish, and were not observed in more than 200 healthy population-matched controls. However, the possibility that some or all of these variants are rare polymorphisms cannot be excluded, and further studies are needed to elucidate their functional significance.

In Finland, occurrence of recurrent founder mutations in several cancer susceptibility genes has been successfully utilized to evaluate the impact of these gene defects in extensive sets of patients and families [11,12,20]. Therefore, to further analyze the importance of the *EPHB2 *variants observed in the Finnish patients (I361V and R568W), their frequencies were analyzed among more than 300 Finnish familial CRC and PC patients. No additional carriers were observed. This suggests that the contribution of these rare germline alterations to familial CRC and PC burden in Finland is limited. The UK variant D861N was not observed in additional 40 HPP patients or in 200 healthy UK controls, suggesting as well a possible role for the variant in colorectal tumor susceptibility.

Overall, germline variants that may be associated with the disease phenotype were seen in 3/101 (3.0%) of patients analyzed for the *EPHB2 *gene. Our data are compatible with the results by Oba et al. (2001), who found no *EPHB2 *germline mutations among 50 CRC patients [21]. Therefore, although some possibly disease associated germline *EPHB2 *variants do exist and may play a role in colorectal tumor predisposition, the observed EPHB2 inactivation in CRCs is largely due to other mechanisms, such as promoter hypermethylation that has frequently been observed in both MSI and MSS tumors, LOH, and somatic mutations in a coding region repeat sequence in MSI colorectal tumors [6,18].

While EPHB2 is the prominent ephrin receptor in the intestine [17], also other members of the protein family may have a role in colorectal tumorigenesis. For example, a dramatic 94-fold decrease in EPHA6 expression has been observed in colorectal tumors when compared to normal tissue, and EPHA8 expression has been detected only in colon tumor but not in corresponding normal tissue [17]. Furthermore, a pattern of inactivation similar to EPHB2 has been observed for EPHB4 and EPHB3 in colorectal tumors and/or cell lines, respectively [5]. Also somatic mutations in the kinase domain of *EPHA3 *have been identified in colorectal tumors [22]. Further studies are needed to more thoroughly elucidate the possible role of this large protein family in CRC tumorigenesis.

## Conclusion

We report here the first *EPHB2 *germline alterations in patients with colorectal tumors. However, the rarity of such alterations in this and in a previous study [21], together with the sequential loss of EPHB2 expression during colorectal carcinogenesis, suggests a limited role for EPHB2 in CRC predisposition and speaks for the more pronounced role for EPHB2 in tumor progression than in tumor initiation. Notwithstanding, germline *EPHB2 *variants do exist and may be associated with colon tumor predisposition, but further studies including functional analyses are needed to confirm this.

## Abbreviations

CRC colorectal cancer, PC prostate cancer, GC gastric cancer, HP hyperplastic polyp, HPP hyperplastic polyposis, EPHB2 ephrin receptor B2, DHPLC denaturing high performance liquid chromatography, MSS microsatellite stable

## Competing interests

The author(s) declare that they have no competing interests.

## Authors' contributions

AK, PL, RL, SK and LGCC carried out the genetic studies. AK, PV and LAA were responsible for drafting and revising the manuscript. LAA, HJ, J-PM, IPMT, JS and CE were involved in design of the study, providing important intellectual content and acquisition of the study material. All authors read and approved the final manuscript.

## Pre-publication history

The pre-publication history for this paper can be accessed here:



## Figures and Tables

**Table 1:** Selection criteria of the 101 patients screened for germline *EPHB2 *mutations.

**Selection criteria**	**Number of patients**	**Country of origin**
Patients with HPP*	38	
HPP	12	USA
HPP	24	UK
HPP	2	FIN
Patients with CRC* and personal or family history of PC*	63	
CRC in the patient and PC in the family	52	FIN
CRC and PC in the same patient	11	FIN

*HPP = hyperplastic polyposis, CRC = colorectal cancer, PC = prostate cancer.

**Table 2:** Observed *EPHB2 *germline variations in colorectal cancer and hyperplastic polyposis patients.

**Patient**	**Exon**	**nt change**	**aa change**	**Index patient (diagnosis, age at diagnosis, nationality)**	**Family history of cancer**	**LOH**	**Frequency in**		
							
							familial PC*	familial CRC*	**healthy controls**
C713	3	239G>A	R80H	CRC, 73 years, FIN	brother, PC, 60 years father, GC*, 57 years	no	-	1/106	9/281
C112	5	1081A>G	I361V	CRC, 75 years and PC, 73 years, FIN	-	yes	0/164	0/139	0/239
C809	9	1702C>T	R568W	serrated CRC, 69 years, FIN	father, PC, 82 years	no	0/164	0/145	0/279
PAP211	14	2581G>A	D861N	HPP*, 58 years, UK	mother, CRC, 36 years	-	-	0/40^†^	0/200

* CRC colorectal cancer; HPP hyperplastic polyposis; PC prostate cancer; GC gastric cancer.

^† ^frequency in additionally screened 40 UK HPP patients
